# The establishment and development of wound repair discipline in China

**DOI:** 10.3389/fsurg.2022.1046494

**Published:** 2022-10-18

**Authors:** Yuesheng Huang, Xiaobing Fu

**Affiliations:** ^1^ Department of Wound Repair, Institute of Wound Repair and Regeneration Medicine, Southern University of Science and Technology Hospital, Southern University of Science and Technology School of Medicine, Shenzhen, China; ^2^ Research Center for Tissue Repair and Regeneration Affiliated to the Medical Innovation Research Department and 4th Medical Center, PLA General Hospital and PLA Medical College, Beijing, China

**Keywords:** wound repair, discipline, traditional wound treatment disciplines, establishment, development

## Abstract

With the acceleration of population aging and the changes of disease spectrum, the number of wound patients has increased annually in the past 20 years, which has become a major problem in terms of China's medical and health work. To address this challenge, the National Health Commission of China issued a notice in November 2019, requiring qualified medical and health institutions to establish wound repair departments to strengthen the standardized diagnosis and treatment management of various wound patients. This article introduces the establishment process of the wound repair discipline in China, as well as the practice and experience of building a high-level wound repair department in Chinese hospitals, hoping that it can be used for reference by peers.

## The process of establishment of wound repair discipline in China

The treatment of Wounded tissues has been an ancient topic. Even since 1600, ancient Egypt had records about wound treatment. After that, from the 5th century BC to the 4th century BC, Hippocrates, the father of medicine in ancient Greece, expounded the importance of hemostasis and bandaging in wound healing. Moreover, the outbreak of the two world wars in the early 20th century further facilitated the development of trauma surgery. As a branch of wound surgery (1), wound repair has also been rapidly promoted.

In China, the wound repair is not independent and has been subordinated to other surgical disciplines before the release of the notice of the National Health Commission [GYB YH (2019) No. 865] in 2019. However, its history can be traced back to the stone age, when ancient people began to treat wound consciously. In the spring and Autumn period and the Warring States period, surgery of traditional Chinese medicine has been gradually formed. In the Han Dynasty, the traditional Chinese medicine developed rapidly. Hua Tuo, a famous trauma doctor in that period, used the herbal anesthetic Ma Fei San to perform operations on patients with wounds and achieved good results. In Sui Dynasty, General Treatise on the Cause and Symptoms of Diseases proposed that the wounds must be sutured immediately after injury, and the suture should be removed once infection occurred (2). In Song Dynasty, the thought of incision and drainage of abscess was more prevailing than ever before. And in the subsequent historical periods, the concept and technology of wound treatment have been developed incessantly. Since the establishment of the People's Republic of China, wound treatment has been rapidly developed and continuously refined. A number of disciplines related to wound treatment have been established nationwide, such as plastic surgery, repair and reconstruction surgery, burn surgery, microsurgery, and integrated Chinese and Western medicine. The continuous improvement of repair technology and the application of a large number of biotechnology and novel materials have significantly improved the treatment effect.

In the early stage of modern wound treatment in China, the wound patients were treated in multiple departments of the hospital due to the absence of independent specialty. Among them, the burn department achieved remarkable results and had a great international impact. Modern wound repair in China began in the 1990s in terms of discipline system construction. The landmark events are mainly the following three (3). First, in 1992, Li Ao, academician of the Burn Research Institute of the Third Military Medical University, and Shi Jixiang, professor of the burn department of Shanghai Ruijin Hospital, led the research on the mechanism of early burn damage and wound healing, which was supported by the first major medical project of the life science department of the National Natural Science Foundation of China, marking the beginning of the national investment and systematic basic research on wound repair. Second, Professor Lin Jian of Jinan University and his team invented growth factor gel, providing products for biological treatment of trauma. Third, academician Fu Xiaobing began to systematically study the relationship between growth factors and tissue repair and regeneration. In 1991, he published the first monograph on growth factors and wound repair in the world, providing a basis for systematically elucidating the mechanism of tissue repair and regeneration at the cellular, molecular and genetic levels. After that, in 1999, 2005 and 2011, the field of trauma and burn was successively supported by three Projects of National Program for Basic Research and Development (973 projects). All of them classified the basic problems of trauma into two key scientific problems: systemic damage and local damage, the former including stress, ischaemia and hypoxia, infection, and the latter mainly indicated wound repair and regeneration. These projects promoted the further research of wound repair and achieved a series of high-level innovative results. Chinese scholars first reported the important discovery of epidermal cell dedifferentiation in the world, which was evaluated as the fourth mechanism of tissue repair and regeneration by international peers. Also the research on sweat gland regeneration was regarded as a milestone type research internationally. While remarkable progress has been made in basic theory, China has also cultivated and created a strong wound repair team. Many talents have been supported by the National Science Fund for Distinguished Young Scholars. And there were more than 20 scientific and technological achievements above the first prize of the provincial and ministerial level, including more than 10 items of the first and second prizes of the national scientific and technological progress.

Meanwhile, academician Fu Xiaobing systematically carried out a study on the epidemiological changes of the refractory wounds on the human body surface in China. It was found that the main etiology of the refractory wounds in China has changed from trauma (burn and infection), in 1998 to new types of chronic wounds such as diabetes foot in 2008–2018, namely, from trauma type to disease type (4). Moreover, the number of patients with chronic wounds has been increasing year by year, with a variety of types and great difficulty in treatment. It is urgent to carry out specialized treatment. Based on the new characteristics of chronic refractory wound epidemiology changes, Chinese scholars began to systematically introduce the importance and necessity of establishing specialized wound treatment wards in academic conferences and professional journals, and called on qualified hospitals to establish wound repair departments. Since 2008, China's wound healing team has carried out standardized training for wound healing professionals, compiled and published a series of wound healing training materials and reference books, and has trained nearly 20,000 doctors and nurses nationwide. These efforts have changed people's traditional understanding of wound treatment and condensed a new consensus, which played an important role in promoting the construction of wound repair department. Since 2010, wound treatment wards have been established in relative surgical departments in nearly 500 hospitals in China, and several small-scale independent wound repair centers have also been established.

On March 10, 2019, after discussing with professors Huang Yuesheng and Lu Shuliang, academician Fu Xiaobing believed that the time was ripe to apply to the National Health Commission to establish the wound repair discipline as an independent tertiary surgical discipline. Professor Huang Yuesheng was entrusted to draft the report, and academician Fu Xiaobing revised and finalized it. In April 2019, academician Fu Xiaobing, together with 28 academicians and two wound repair experts, Huang Yuesheng and Lu Shuliang, signed the report and officially submitted it to the National Health Commission on May 5. The construction standards of wound repair department and the basic skills requirements of doctors and nurses were submitted in accordance with the requirements of the National Health Commission. On July 9, the National Health Commission sent notice to provincial health administrative departments and relevant first-class societies and associations across the country to solicit opinions on the basic standards for wound repair departments of medical and health institutions (for Trial Implementation) and the basic skills requirements for clinicians and nurses in wound repair departments (for Trial Implementation). In the middle of September, the expert group received the feedback from the medical administration and hospital administration of the National Health Commission, and then revised, supplemented and improved the construction standards of wound repair department and the basic skills requirements of doctors and nurses.

On November 29, 2019, the general office of the National Health Commission issued a notice on strengthening the management of the diagnosis and treatment of chronic and refractory wounds on the body surface [(2019) No. 865]. The notice requires health administrative departments at all levels and various medical institutions in the country to attach great importance to the management of the diagnosis and treatment of chronic refractory wounds on the body surface, and also requires medical and health institutions with conditions to establish wound repair departments (5). The National Health Commission Capacity Building and Continuing Education Center quickly established an expert committee of wound repair department responsible for the training of wound repair doctors and nurses. So far, the establishment process of wound repair discipline as a new three-level surgical discipline in China has been completed. And the construction of wound repair specialized departments has been started in all parts of the country.

## Practice and experience in the construction of high-level wound repair discipline in China

The notice of the general office of the national health and Health Commission (2019) No. 865 on strengthening the management of the diagnosis and treatment of chronic refractory wounds on the body surface stipulates that the wound repair department is a new independent surgical tertiary discipline and requires qualified medical institutions to establish a wound repair department. The notice provides a basic basis for the construction of the wound repair department and opens an era of the construction of the wound repair discipline. More than 2 years after the notice was issued, about 400 hospitals in China are preparing to establish wound repair departments and dozens of wound repair departments have already been established. China's practices and experiences on how to accelerate the construction of high-level wound repair discipline are as follows.

### Enlisting the support of the hospital

The prevention and control of chronic refractory wounds has become a major challenge for the country, which affects the smooth implementation of the healthy China strategy to a certain extent. The increasing demand for clinical treatment urgently calls for the establishment of wound repair discipline. Under this circumstance, the National Health Commission, in response to the new situation of changes in the disease spectrum and the treatment needs of wound patients, timely issued the notice on strengthening the diagnosis and treatment management of chronic refractory wounds on the body surface, requiring medical and health institutions with conditions to establish wound repair departments, which opened a new era of wound repair discipline construction (6).

As an emerging discipline, the key to realize the independent and high-level establishment of wound repair is whether the health administrative departments and hospitals attach enough importance to it. In China, the construction of wound repair discipline has just started, and each hospital is basically on the unified starting line. If the hospital can seize the opportunity to build a high-level wound repair specialty and actively use various channels and media for publicity at the same time, it will be easy to seize the opportunity in all aspects. The promising future prospect of this discipline can also increase confidence and determination in the construction of wound repair department in the hospital.

There are many causes and types of chronic wounds. Due to historical reasons, such patients are often treated in nearly 10 different departments of the hospital, forming an embarrassing situation that “all are treated, but not standard and the best treatment” (7). Therefore, at the beginning of the establishment of the wound repair department, contradictions are inevitable with the traditional disciplines due to the blurry admission scope. And it is necessary for the hospital to define the definite scope of admission of the wound repair department within the hospital according to the requirements of the National Health Commission, and coordinate the relationship between various relevant disciplines to reduce conflicts. In addition, the wound repair department is a new discipline, which is still very weak. Many treatments have not been included in the medical insurance for the time being, and these problems need to be coordinated and solved by relevant state departments and hospitals. As long as they attach great importance to it, these problems will be solved smoothly with the development of wound repair discipline and the continuous improvement of treatment level.

### Strengthening the construction of talents

A strong talent team is the key to building high-level wound repair discipline. It is of great need to train wound repair specialized doctors and nurses nationwide according to the skill requirements in the notice of the National Health Commission. Therefore, wound repair skills training classes for directors, doctors and nurses respectively have been held regularly by the expert committee of wound repair department in China. Moreover, on the basis of the constructing research-based disciplines, sufficient number of research-based clinicians and high-quality nursing personnels who can carry out nursing research are also being trained according to the professional standards of doctors and nurses (8, 9). Since patients with chronic wounds usually suffer from basic diseases, for these wound repair department with a large scale of patients or with a large quantity of a certain type, doctors from relative clinical departments can be invited to assist in the treatment of patients. For example, the endocrinology department can be asked to manage the basic diseases of patients with diabetes foot to control blood sugar (10).

To establish a strong talent team, firstly, it is necessary to cultivate excellent academic leaders, who should have the following qualities: Good clinical and scientific research ability; Cohesion and centripetal force; Sense of mission, regarding discipline construction as a mission and taking responsibility; International vision, building disciplines from an international or broader perspective; Innovative consciousness, being able to grasp the frontier of the discipline and find a breakthrough point on the basis of mastering the dynamics of the discipline. Secondly, efforts should be made to train a number of outstanding academic backbones, each of whom has its own research direction and objectives; Thirdly, a team of research-oriented clinicians and nurses should be trained with reasonable structure and echelon to lay a talent foundation for the sustainable development of the discipline.

### Strengthening support conditions

Support conditions are the basis and premise for discipline construction. Hospitals that intend to establish wound repair departments need to follow the basic standards of wound repair departments of medical institutions issued by the National Health Commission, and configure necessary medical equipment and facilities according to the actual situation and treatment needs of the unit. In hospitals where the conditions are permitted, first-class wound repair facilities and basic research platforms for research should be built according to high standards. What's more, a certain amount of funds should be invested yearly to update and upgrade the research platform to ensure advanced and supporting conditions to meet the needs of innovative researches (11).

### Vigorously facilitating specialized technology

The high-level wound repair discipline depends on the high-level wound repair innovative clinical technology and the high-level wound repair innovative scientific research achievements. It should endeavour to promote new clinical technologies and scientific research with the goal of building a high-level and research-oriented wound repair discipline. According to the clinical needs and development frontiers, three directions of wound repair discipline are determined as follows: (a) Clinical technology of wound repair, including inventing wound repair and rehabilitation technology with potential clinical application prospect, formulating national expert consensus, guidelines, specifications, clinical paths, standards, etc., and standardizing wound repair and treatment behavior (12–20); (b) Wound microenvironment and repair: technologies and measures to create a microenvironment suitable for wound repair, the microenvironment refers to oxygen, bioelectric field, inflammation and immunity, temperature, pH, extracellular matrix, stem cells and promoting healing factor; (c) Invention, research and transformation of new types of wound repair materials, these biomaterials can be classified into four categories. One can build a suitable microenvironment for wound healing, one can load with stem cells and various healing promoting factors (growth factors, exosomes, etc.), novel functional and intelligent biomimetic wound repair materials with anti-infective agents (21–30).

### Coordinating the relationship with traditional wound treatment disciplines

Due to the historical reasons, wound patients are often treated dispersedly in different departments of the hospital, including burn department, orthopedics department, hand surgery department, vascular surgery department, general surgery department, traditional Chinese medicine department, dermatology department, endocrinology department and wound care nursing center. They are also collectively referred to as traditional wound treatment disciplines. However, in order to treat all kinds of chronic wound patients in a centralized and standardized manner, so as to carry out specialized treatment and improve treating effect, it is now necessary to establish the wound repair department, an independent three-level surgical discipline. Some experts in traditional wound treatment are worried that the establishment of wound repair department will affect the construction and development of traditional disciplines. Therefore, eliminating this kind of concern is an important prerequisite for the smooth construction of the wound repair discipline. On this basis, the construction team of the wound repair discipline in China elaborated the differences and links between the wound repair discipline and the traditional wound treatment discipline from many aspects, including prevention and control, occurrence mechanism and discipline system, so as to make them develop in a coordinated manner. The wound repair discipline should conform to the requirements of discipline connotation, strive for characteristic development, learn from and cooperate with traditional wound treatment disciplines, and seek common development, so that the traditional wound treatment discipline can support the construction of wound repair department.

### Meeting the challenges and striving to construct wound repair discipline

As a new surgical discipline, there are still many problems and difficulties in the construction of wound repair. Above all, ideological understanding concerning on wound repair must be deepened. Relevant leaders and experts need to fully understand the importance of the construction of wound repair discipline, thus to vigorously support and promote the relating construction, such as the space (wards), personnel, funds, equipment and facilities. As a new discipline, wound repair contains both opportunities and challenges. However, it is necessary to build the wound repair discipline under the pressure of various difficulties. By winning the attention and support of leaders, leaders should possess a strategic vision, considering the new discipline as a new growth point of the hospital discipline construction, and aim at the future. Though solvation of the problems that constantly arises during the process of discipline construction, the discipline will be well constructed.
